# Energy materials: supramolecular nanoparticles for solar energy harvesting

**DOI:** 10.3402/nano.v4i0.21079

**Published:** 2013-06-11

**Authors:** E. S. Shibu, A. Sonoda, Z. Tao, Q. Feng, A. Furube, S. Masuo, L. Wang, N. Tamai, M. Ishikawa, V. Biju

**Affiliations:** 1 Health Research Institute, National Institute of Advanced Industrial Science and Technology (AIST), Takamatsu, Japan; 2 Department of Advanced Materials Science, Kagawa University, Takamatsu, Japan; 3 Research Institute for Instrumentation Frontier, AIST, Tsukuba, Japan; 4 Department of Chemistry, Kwansei Gakuin University, Sanda, Japan; 5 PRESTO, Japan Science and Technology Agency, Tokyo, Japan

## SNPs form powerful and durable antenna systems for solar cells

Renewable resource of carbon-free energy is crucial in the current scenario of global warming and our growing energy needs. Besides, the significance of safer energy resources has been underscored by many nuclear mishaps, including the recently crippled Fukushima Nuclear power plant in Japan. Sunlight is the most promising alternative to nuclear fuels and greenhouse-gas-emitting fossil and alcohol fuels. A combination of the unique optical properties of quantum dots (QDs) such as broad absorption, exceptionally high values of molar extinction coefficient, and uncompromised photostability with the multi-electron acceptor fullerene offers exciting prospects for light energy harvesting. V. Biju, E. S. Shibu and co-workers (1) from AIST-Shikoku in Japan have now demonstrated the photofabrication of fullerene-shelled QD supramolecular nanoparticles (SNPs), in which the fullerene shell acts as a well-defined electron acceptor and also as a robust protecting layer against the photocorrosion of the QD core by iodine in the I^−^/${\text{I}}_{3}^{-}$ redox couple – ‘breakthrough nanomaterials for solar energy harvesting,’ says Biju.

Researchers have synthesized novel fullerene-shelled QD SNPs by the covalent tethering of a fullerene-thiol monolayer to the QD followed by the UV (256 nm)-induced [2+2] cyclo-addition reactions of free fullerenethiol to the tethered monolayer. Scanning electron micrograph and atomic force microscopy image show highly uniform SNPs. Here, the correlated single-photon emission and the two-state ON–OFF photoluminescence studies show single molecule behavior of SNPs. The fullerene-shells suppress the blinking of single QDs by acting as well-defined electron traps, without allowing the transfer of Auger electrons to unknown traps. Electron transfer from the QD-core to the fullerene-shell is apparent from the short ON and OFF durations in the photoluminescence intensity trajectories of single QDs, quenching of the photoluminescence intensity and lifetime of QDs at the ensemble level, and the characteristic transient absorption band of the anion radical of fullerene.

**Figure:**
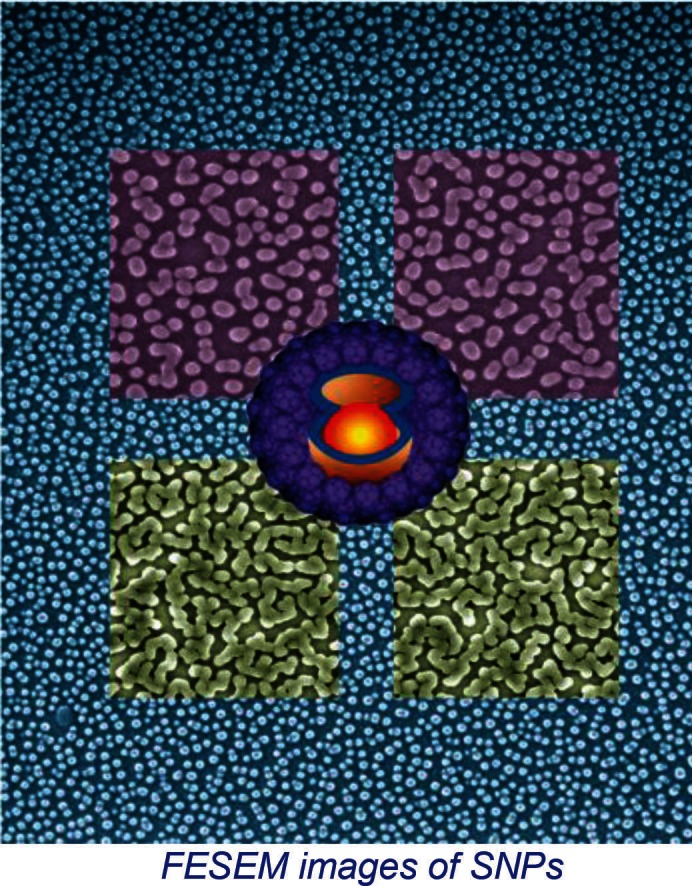


Using these unique nanomaterials, the researchers have constructed a photoelectrochemical cell and the transferred electron has been externally driven under simulated sunlight of AM 1.5 (100 mW/cm^2^). The new photoelectrochemical cell composed of SNPs is able to produce a photocurrent of 400 µA/cm^2^ with an open circuit voltage of 37 mV, which is truly better than the performance by fullerene-QD dyad systems.

Here, the electron transfer from the highly stable QD to the protecting fullerene-shell places the SNPs among the most promising antenna systems for solar energy harvesting. Now, researchers are looking for the improvisation of the SNPs in some way, so as to produce larger photocurrent response. Nevertheless, the fullerene-shell offers physical and chemical stability and biosafety to the core QD.
